# The Impact of a 24-h Low and High Fermentable Oligo- Di- Mono-Saccharides and Polyol (FODMAP) Diet on Plasma Bacterial Profile in Response to Exertional-Heat Stress

**DOI:** 10.3390/nu15153376

**Published:** 2023-07-29

**Authors:** Stephanie K. Gaskell, Kayla Henningsen, Pascale Young, Paul Gill, Jane Muir, Rebekah Henry, Ricardo J. S. Costa

**Affiliations:** 1 Department of Nutrition Dietetics & Food, Monash University, Notting Hill, VIC 3168, Australia; stephanie.gaskell@monash.edu (S.K.G.); kayla.henningsen@monash.edu (K.H.); pascale.young@monash.edu (P.Y.); 2 Department of Gastroenterology, Monash University, Melbourne, VIC 3004, Australia; paul.gill@monash.edu (P.G.); jane.muir@monash.edu (J.M.); 3 School of Public Health and Preventive Medicine, Monash University, Clayton, VIC 3168, Australia; rebekah.henry@monash.edu; 4 Department of Civil Engineering, Monash University, Clayton, VIC 3168, Australia

**Keywords:** running, thermoregulation, I-FABP, endotoxin, bacteremia, inflammatory cytokines, gastrointestinal symptoms, breath hydrogen, short chain fatty acids

## Abstract

Exertional-heat stress (EHS) compromises intestinal epithelial integrity, potentially leading to the translocation of pathogenic agents into circulation. This study aimed to explore the impact of EHS on the systemic circulatory bacterial profile and to determine the impact of a short-term low (LFOD) and high (HFOD) fermentable oligo- di- mono-saccharide and polyol dietary intervention before EHS on this profile. Using a double-blind randomized cross-over design, thirteen endurance runners (*n* = 8 males, *n* = 5 females), with a history of exercise-associated gastrointestinal symptoms (Ex-GIS), consumed a 24 h LFOD and HFOD before 2 h running at 60% $\dot{V}$O_2max_ in 35.6 °C. Blood and fecal samples were collected pre-EHS to determine plasma microbial DNA concentration, and sample bacteria and short chain fatty acid (SCFA) profiles by fluorometer quantification, 16S rRNA amplicon gene sequencing, and gas chromatography, respectively. Blood samples were also collected post-EHS to determine changes in plasma bacteria. EHS increased plasma microbial DNA similarly in both FODMAP trials (0.019 ng·μL^−1^ to 0.082 ng·μL^−1^) (*p* < 0.01). Similar pre- to post-EHS increases in plasma *Proteobacteria* (+1.6%) and *Firmicutes* (+0.6%) phyla relative abundance were observed in both FODMAP trials. This included increases in several *Proteobacteria* genus (*Delftia* and *Serratia*) groups. LFOD presented higher fecal *Firmicutes* (74%) and lower *Bacteroidota* (10%) relative abundance pre-EHS, as a result of an increase in *Ruminococcaceae* and *Lachnospiraceae* family and respective genus groups, compared with HFOD (64% and 25%, respectively). Pre-EHS plasma total SCFA (*p* = 0.040) and acetate (*p* = 0.036) concentrations were higher for HFOD (188 and 178 μmol·L^−1^, respectively) vs. LFOD (163 and 153 μmol·L^−1^, respectively). Pre-EHS total fecal SCFA concentration (119 and 74 μmol·g^−1^; *p* < 0.001), including acetate (74 and 45 μmol·g^−1^; *p* = 0.001), butyrate (22 and 13 μmol·g^−1^; *p* = 0.002), and propionate (20 and 13 μmol·g^−1^; *p* = 0.011), were higher on HFOD vs LFOD, respectively. EHS causes the translocation of whole bacteria into systemic circulation and alterations to the plasma bacterial profile, but the FODMAP content of a 24 h diet beforehand does not alter this outcome.

## 1. Introduction

There is now compelling evidence that exercise perturbs various integrity and functional aspects of the gastrointestinal tract and subsequently prompts systemic immune responses [1,2]. Termed ‘*Exercise-Induced Gastrointestinal Syndrome*’ (EIGS), its etiology and pathophysiology, and links to exercise-associated gastrointestinal symptoms (Ex-GIS), have previously been comprehensively described and recently updated elsewhere [3,4,5,6]. Accumulated evidence now supports the concept that prolonged strenuous exercise in hot ambient conditions (i.e., exertional-heat stress (EHS)), resulting in marked thermoregulatory strain, promotes greater gastrointestinal disturbance compared with other EIGS extrinsic exacerbating factors, such as exercise intensity and modality [7,8,9,10,11,12,13,14,15,16,17,18,19,20]. For example, 2 h of steady state (e.g., 60% $\dot{V}$O_2max_) running in ≥35.0 °C, with peak core body temperature achieving ≥39.0 °C, consistently results in greater intestinal epithelial injury, intestinal hyperpermeability, disturbed endotoxin profile (i.e., gram-negative bacterial endotoxin, endogenous endotoxin core antibodies, sCD14, and/or lipopolysaccharide binding protein) and systemic inflammatory response (SIR) profiles, and Ex-GIS compared with 2 h running at <35.0 °C, with peak core body temperature < 39.0 °C, even with the application of high exercise intensities (≥70% $\dot{V}$O_2max_), with or without short exercise durations, as reported in Costa et al. [3].

Disturbances to gastrointestinal integrity as a result of exercise are generally asymptomatic and/or cause minor inconvenience in most cases, but they may also lead to severe Ex-GIS that compromise performance, and/or clinically serious issues necessitating medical attention and intervention (e.g., acute reversable colitis, gastroparesis, paralytic ileus, abnormal stools, and/or projectile vomiting) in a minority of cases [6,21,22,23,24,25,26]. In some rare cases, it may cause fatality in the presence of prerequisite exacerbation factor exposure (e.g., sepsis and SIRS, in the pathophysiology of heat stroke) [27,28,29,30]. The mechanisms by which EIGS may lead to fatality lie with the translocation of pathogenic agents (e.g., bacteria and/or bacterial endotoxins) from the gastrointestinal lumen through local intestinal epithelial and hepatic tissues, and subsequent access into systemic circulation. Such pathogenic translocation may instigate an over-exaggerated systemic immune response (e.g., in response to sepsis) that may ultimately lead to refractory shock and multi-organ failure [31]. Research into exercise-associated endotoxemia has been thoroughly investigated, with strengths and limitations in methodologies previously described (e.g., LAL endpoint assay, EndoCAb exploration, and/or experimental design issues) [1,3,31,32]. However, less is known about the magnitude of systemic bacteremia (i.e., the translocation of whole bacteria from the lumen into circulation) that exercise may induce. Although the clinical identification of whole bacteria in blood is a known assessment practice in cases of sepsis, using culture or 16S rRNA amplicon sequencing techniques [33], researching and practising these assessment techniques in exercise are not common. Nevertheless, a recent study confirmed a significant pre- (0.03 ng·μL^−1^) to post-exercise (0.07 ng·μL^−1^) increase in plasma microbial DNA concentration. This was accompanied by an increase in plasma *Proteobacteria* (+7.9%), which included *Proteobacteria* genus groups (i.e., *Sandarakinorhabdus*, *Sphingomonas*, *Polaromonas*, *Massilia*, *Aquabacterium*, and *Providencia*), and a decrease in *Firmicutes* (−4.2%), as well as *Cyanobacteria* (−2.9%), phyla relative abundance, which included a reduced plasma bacteria α-diversity in response to a 2 h high intensity interval training exercise protocol [20]. These combined outcomes suggest a state of exercise-associated bacteremia, likely due to the lumen originating translocation of whole bacteria into systemic circulation resulting from exercise linked compromised intestinal epithelial integrity. However, the impact of a substantial EHS load on markers of exercise-associated bacteremia has not yet been explored [3], and it is likely to result in greater bacterial profile outcomes compared with previous exertional-only experimental protocols [12,20].

Considering the potential clinical implications of EIGS, it is therefore not surprising that more research is being undertaken to understand and establish effective EIGS prevention and management strategies [3]. One such strategy that is gaining interest is the manipulation of the gastrointestinal microbial composition via dietary intervention in order to increase the relative abundance of short chain fatty acid (SCFA, e.g., acetate, butyrate, propionate)-producing commensal bacteria and decrease the relative abundance of potent pathogenic bacteria and associated endotoxins [7,34]. It is suggested that dietary manipulation, such as increasing fermentable carbohydrates that principally act as a prebiotic, may subsequently lead to increased SCFA commensal bacterial diversity and SCFA luminal concentration [35,36,37]. Such alterations in luminal composition may provide a protective mechanism to intestinal epithelial integrity (e.g., luminal anti-microbial protein secretions and mucus production, enterocyte cell proliferation, tight-junction protein expression, reduced pathogenic adherence to intestinal epithelial apical surface) and systemic responses, although the focus has been primarily on clinical populations [38,39,40,41]. However, such benefits have not been confirmed in healthy active and/or athlete populations, with or without exercise-associated compromise to gastrointestinal status. Although gastrointestinal microbial profile in athletes has been previously studied [42], research outcomes have been heterogeneous and with no normative values established, likely associated with differing athlete populations and dietary patterns, methodological issues, and/or varying degrees of confounder experimental control [3]. It is also important to note that such athlete ‘gut microbiome’ exploration has scarcely determined SCFA concentrations in adjunct. Therefore, there is a lack of comprehensive investigation in exploring fecal and plasma bacterial composition and SCFA concentration collectively, in conjunction with EIGS and Ex-GIS. Nevertheless, a recent study showed robust test–retest values for total and differential fecal and plasma SCFA concentration and an association between pre-exercise concentration and EIGS gastrointestinal–circulatory pathway biomarkers [20].

SCFA appear to be at the centre for possibly inducing a positive effect on gastrointestinal integrity and systemic immune responses (e.g., immunomodulation) to cope with exercise-associated endotoxemia and/or bacteremia. Considering that fermentable oligo- di- mono-saccharides and polyols (FODMAP) are characterized by their prebiotic properties and can be used by luminal microbial agents (i.e., bacteria) to produce SCFA as a result of fermentation processes [43,44,45], their manipulation may act as a primary management strategy for EIGS and Ex-GIS [6,11,46,47]. In a recent study comparing a 24 h low (2 g·day^−1^) versus high (47 g·day^−1^) FODMAP diet, exercise-associated intestinal epithelial injury was significantly less, and there was lower disturbance to endotoxin profile, in response to the EHS, on the high FODMAP diet versus the low FODMAP diet [11]. It was proposed that the high FODMAP diet ameliorating effects on gastrointestinal integrity markers were due to: (i) malabsorbed carbohydrate and fermentation in the lumen promoting villi microvascular perfusion, as observed in Snipe et al. [17], with continuous carbohydrate feeding during exercise; and/or (ii) increased SCFA concentration associated with the consumption of fermentable carbohydrates, even considering the short-term dietary intervention [43,44,45]. Although there is repeated evidence that small and frequent carbohydrate intake during exercise can maintain intestinal epithelial integrity, likely due to maintained villi microvascular perfusion [17,48,49,50,51,52], little is known to date about the impact of pre-exercise SCFA lumen and/or plasma concentration on EIGS and Ex-GIS [20].

With these background points in mind, the aims of the current study were two-fold: first to explore the impact of EHS on the magnitude and profile of circulatory bacteria, and second to determine the impact of a short-term low and high FODMAP dietary intervention on the magnitude and profile of circulatory bacteria in response to EHS. It was hypothesised that EHS will induce bacteremia and increase the relative abundance of *Proteobacteria* bacterial groups in plasma. It was also hypothesised that HFOD would result in lower EHS-induced bacteremia, as a result of higher pre-EHS faecal and plasma total and differential SCFA concentrations.

## 2. Methods

### 2.1. Participants

The current study is an extended sample analysis of previously published research, which describes in full the experimental procedures [11]. Eighteen non-heat acclimatized recreationally competitively trained endurance and ultra-endurance runners volunteered and initiated participation in the original study. Of these, additional sample availability and further analysis was undertaken in thirteen participants [mean ± SD (*n* = 8 males and *n* = 5 females): age 34 ± 7 years, body mass (BM) 67.7 ± 10.3 kg, height 1.75 ± 0.08 m, percentage of body fat mass 18 ± 6%, and maximal oxygen uptake ($\dot{V}$O_2max_), 63.9 ± 9.7 mL‧kgBM^−1^‧min^−1^]. Prior to starting the experimental procedures, all participants gave informed consent. The local ethics committee (MUHREC: 8847) approved the original study, which conformed to the Helsinki Declaration for Human Research Ethics. The standard exclusion criteria and participants’ familiarity with FODMAPs have previously been described [11]. All participants reported previous incidence of Ex-GIS during training and/or competition.

### 2.2. Experimental Procedures

**Figure 1:**
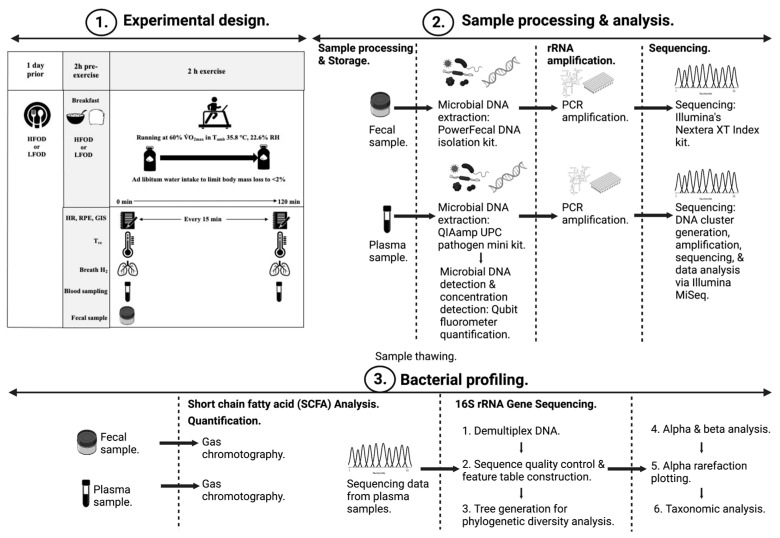
Schematic description of experimental and sample analytical procedures.

A week prior to the first experimental trial, participants undertook preliminary testing that included anthropometrical measures (i.e., height, BM, and body composition). Participants also undertook a maximal exercise test to volitional exhaustion on a motorized treadmill (MyRun Technogym; Technogym, Cesena, Italy) to establish $\dot{V}$O_2max_ and respective running speed at 60% $\dot{V}$O_2max_ for the main experimental trials (11.2 ± 1.7 km‧h^−1^). A schematic illustration describing the experimental procedures has been previously reported in Gaskell et al. [11], and further sample analytical methods are described in Figure 1. Utilising a computer-generated double-blind randomised crossover design, participants were provided with a high (HFOD) [energy 2762 ± 844 kcal‧day^−1^, protein 104 ± 32 g‧day^−1^, carbohydrate 409 ± 139 g‧day^−1^, fat 70 ± 19 g‧day^−1^, fibre 54 ± 17 g‧day^−1^, total FODMAP 51 ± 30 g‧day^−1^] or low (LFOD) [energy 2503 ± 640 kcal‧day^−1^, protein 92 ± 26 g‧day^−1^, carbohydrate 352 ± 94 g‧day^−1^, fat 68 ± 17 g‧day^−1^, fibre 50 ± 11 g‧day^−1^, total FODMAP 2 ± 1 g‧day^−1^] FODMAP diet for a 24 h period before each respective experimental trial. Dietary development, control processes, blinding, and dietary compliance have been previously reported in Gaskell et al. [11]. Matching the 24-h FODMAP diet (i.e., HFOD or LFOD), a pre-exercise breakfast was consumed 2 h before the initiation of the EHS, and a recovery beverage was consumed immediately post-EHS. Dietary interventions were separated by a 1-week washout period. Participants refrained from strenuous exercise for 48 h before each trial.

On the day of each main experimental trial, participants reported to the laboratory at 8:00 a.m., after consuming their assigned breakfast at 7:00 a.m. Participants were asked to void before nude BM measurement. During voiding, participants were asked to provide a ~30 g mid-flow fecal sample into a sterilised fecal collection container (SARSTEDT Australia Pty Ltd., Mawson Lakes, South Australia, Australia), which was immediately stored frozen at −80 °C until analysis. Blood samples were then collected by venepuncture from an antecubital vein into lithium heparin (6 mL, 1.5 IU‧mL^−1^ heparin) vacutainers. Rectal temperature (T_re_) was monitored during running, with participants inserting a thermocouple 12 cm beyond the external anal sphincter (Grant Squirrel data logger, Shepreth, UK). Participants then completed a 2 h (initiated at 9:00 a.m.) pre-determined running exercise bout within an environmental chamber in hot ambient conditions (HFOD: 35.6 ± 0.6 °C ambient temperature and 23.0 ± 1.8% relative humidity (RH), and LFOD: 35.6 ± 0.3 °C and 23.7 ± 3.5%; with dual fan wind speed 10.6 km‧h^−1^). Water was provided *ad libitum* (HFOD 764 ± 357 mL‧h^−1^ and LFOD 692 ± 265 mL‧h^−1^) during EHS for autonomy of drinking patterns to minimise the programmed drinking-induced occurrence of Ex-GIS [8,53]. T_re_, heart rate, Borg scale (6–20) rating of perceived exertion (RPE), 13-point McGinnis thermal comfort rating (TCR), and BM were measured and recorded every 15 min during EHS [18,19]. Immediately after exercise, blood samples were collected and nude BM was measured. Immediately after these, participants consumed a HFOD or a LFOD recovery beverage containing 1.2 g‧kgBM^−1^ of carbohydrate and 0.4 g‧kgBM^−1^ of protein, corresponding to their 24 h FODMAP diet trial, as per recovery optimization guidance [54]. Participants remained seated during the 4 h recovery period and consumed water ad libitum, as per the primary experimental procedures [11]. To reduce any seasonal heat acclimatization [55], experimental trials were conducted over the cooler seasonal periods (ambient temperature consistently ≤20 °C).

### 2.3. Blood Sample Processing and Analysis

Blood hemoglobin (Hemocue Hb 201+, HemoCue, Angelholm, Sweden) and hematocrit (capillary method) were determined to estimate plasma volume change and used to correct plasma variables relative to baseline [3]. The remaining heparin blood samples were centrifuged at 4000 rpm (1500× *g*) for 10 min within 15 min of sample collection. Plasma was aliquoted into sterile microstorage tubes (Axygen, Corning Incorporated, Reynosa, Mexico) and frozen at −80 °C until analysis. However, two 50 μL heparin plasma aliquots were used to determine plasma osmolality (P_Osmol_) in duplicate (CV: 2.1%) by freezepoint osmometry (Osmomat 030; Gonotec, Berlin, Germany).

### 2.4. Plasma and Fecal Bacterial Profiling

Microbial DNA was extracted from plasma samples using a QIAamp UPC pathogen mini kit (Qiagen, Germantown, TN, USA). After thawing, 200 μL of heparin plasma was added to 1.5 mL glass microbead tubes and underwent mechanical lysis in accordance with the manufacturer’s instructions. Thereafter, 400 μL of the pre-treated sample underwent the spin protocol for chemical lysis in accordance with the manufacturer’s instruction. Purified extracted DNA (100 μL sample) was immediately frozen at −20 °C prior to bacterial profiling. To avoid artefact outcomes due to low detection levels of microbial DNA in plasma and the high risk of contamination, standardized control procedures were implemented throughout the sample collection, processing, and analysis [56,57]. Samples were processed using sterile consumables and in a biohazard ventilation cabinet. Microbial DNA detection and concentration was performed by Qubit fluorometer quantification (ThermoFisher Scientific, Waltham, MA, USA), with minimal detection set at 0.05 ng/mL. Blank control (pyrogen/RNAse/DNA/se free water) samples yielded undetectable outcomes. Extracted DNA was delivered to the Micromon Next Generation Sequencing Facility (Monash University, Clayton, Australia) for the PCR amplification of the V3-V4 region of the 16S rRNA gene as previously described [7]. The assembled reads were analysed using QIIME2 (v.2019.1), as previously described [7]. Briefly, reads were imported into QIIME2, and quality assessment, filtering, barcode trimming, and chimera detection were performed using the DADA2 pipeline [58]. Taxonomic evaluation was conducted using pre-set parameters (98% identify, confidence *p* < 0.05%) with the SILVA 138.1 release [59].

For fecal samples, upon thawing at room temperature and homogenisation, 0.20–0.30 g of each sample was transferred to a 2 mL dry garnet bead microtube, before the addition of bead solution. Cell lysis, sample purification, and DNA extraction were then performed as per manufacturer’s instructions (PowerFecal DNA isolation kit, Qiagen, Germantown, TN, USA), using sterile consumables and in a biohazard ventilation cabinet. Blank control samples, using pyrogen/DNAse/RNAse free water, were run simultaneously in duplicate. Purified extracted DNA (50 μL sample) was immediately frozen at −20 °C prior to bacterial gene sequencing. Extracted genomic DNA was delivered to the Australian Genome Research Facility (Melbourne, Australia) for the PCR amplification of the V3–V4 region of the 16S rRNA gene and sequencing on the Illumina MiSeq platform utilising the Illumina’s Nextera XT Index kit. Blank control samples yielded undetectable outcomes.

Before statistical analysis, sequencing data for phyla, family, and genus amplicon sequence variants were calculated by dividing the number of reads for each taxon by the number of reads in the faecal and plasma samples, taking into consideration background counts detected in blank control samples. 16S rRNA sequences per faecal and plasma samples ranged from 54,009 to 214,016 and from 11,581 to 64,582, respectively. To avoid the risk of including artefact values in data analysis, resulting from potential contamination during sample handling (i.e., sample collection, processing, and analysis procedures), for amplicon sequence variants (AVS), only bacterial groups with ≥0.5% relative abundance, respective to the determination medium, were included for data analysis. Bacterial calculations of *n* = 5 and *n* = 5 phyla, *n* = 14 and *n* = 20 family, and *n* = 11 and *n* = 40 genus AVS for plasma and fecal samples, respectively, were adequately detected for relative abundance (≥0.5%) and α-diversity determination (i.e., Shannon Equitability Index (SEI)).

### 2.5. Plasma and Fecal SCFA Analysis

Plasma samples (heparin) were analysed in duplicate for SCFA content using gas chromatography, as previously described [59]. Briefly, 300 μL of plasma was spiked with 50 μL of 200 μM heptanoic acid and acidified with the addition of 10% sulfosalicylic acid. SCFA were extracted using a diethyl ether solvent, with the organic layer transferred into alkaline 0.2 M NaOH. The alkaline solution was concentrated by nitrogen evaporation, dissolved in 1 M phosphoric acid, and transferred into a cold GC glass vial for analysis using an Agilent GC6890 coupled to FID, with helium used as the carrier gas. An Agilent FFAP column (30 m × 0.53 mM (internal diameter) × 1.00 μM (film thickness)) was used for analysis. A splitless injection technique was used, with 0.2 μL of sample injected. Upon injection, the oven was held at 90 °C for 1 min, increased to 190 °C at 20 °C/min, and held for 3 min. Concentrations for acetate, propionate, and butyrate were calculated from the average of triplicate analysis, where the CV was <20%. Total SCFA was calculated by the sum of the individual SCFA. Results were expressed as μmol/L.

Fecal SCFA was quantified using gas chromatography as previously described [60]. Thawed faecal material was spiked with internal standard (1.68 mM heptanoic acid), homogenized, and centrifuged (2000× *g*, 10 min, 4 °C). After centrifugation, supernatant was filtered through a 0.2 μm filter vial containing 1 M phosphoric acid. Samples were analysed using an Agilent GC6890 as described above. A CV <10% within triplicate samples was used as a quality control measure. 

### 2.6. Statistical Analysis

The confirmation of adequate statistical power *a priori* for the primary research has been previously described [11]. Post hoc, based on the statistical test, mean, SD, and effect size, and applying a standard alpha (0.05) and beta value (0.80), the current participant sample size is estimated to provide adequate statistical power (power* 0.80–0.99) for detecting significant exercise and dietary associated differences within and between groups (G*Power 3.1, Kiel, Germany). Descriptive data in the text are presented as mean ± SD, and for primary and secondary variables as mean and 95% confidence interval (CI), as indicated. Data in figures are presented as relative abundance (%) of total identified bacterial presence or mean ± SEM for clarity, where indicated. Prior to the application of comparative statistical tests, diagnostic checks (Shapiro–Wilks test of normality and Levene’s homogeneity of variance) were performed. Comparison data were examined using paired-sample t-test or Wilcoxon where applicable. Additionally, Cohen’s standardised measurement of effect size was applied where appropriate and was determined as *d* = 0.50 and *d* = 0.80 for medium and large effects, respectively. Full sample collection and analysis was not possible for all recruited participants. Therefore, only participants with full data sets for each sample variable were used in data analysis, with participant numbers reported in the respective figures and tables. Statistics were analysed using SPSS (V.27.0, Chicago, IL, USA) with significance accepted at *p* ≤ 0.05.

## 3. Results

### 3.1. Exertional-Heat Stress (EHS) and EIGS Markers

No difference (*p* > 0.05) in markers of physiological [(mean and 95% CI) Δ 15–120 min HR: HFOD 20 (12 to 16) bpm and LFOD 14 (10 to 13), and Δ 15–120 min RPE: HFOD 2 (2 to 3) and LFOD: 2 (1 to 2)] or thermoregulatory strain [maximal T_re_: HFOD 39.0 (38.6 to 39.3) °C and LFOD 39.0 (38.5 to 39.4) °C, and Δ 15–120 min T_re_: HFOD 1.4 (1.0 to 1.7) °C and, and LFOD 1.6 (1.2 to 1.9) °C, Δ 15–120 min TCR: HFOD 1 (1 to 2) and LFOD 1 (1 to 1)] were observed between the two FODMAP dietary trials in response to EHS. No difference in pre- and post-EHS blood glucose responses [HFOD 4.6 (4.2 to 4.9) mMol·L^−1^ and 5.9 (5.6 to 6.3) mMol·L^−1^, and LFOD 4.6 (4.3 to 4.8) mMol·L^−1^ and 5.7 (5.4 to 6.0) mMol·L^−1^, respectively], pre- and post-exercise hydration status [P_Osmol_ HFOD 296 (293 to 298) mOsmol·kg^−1^ and 297 (294 to 300) mOsmol·kg^−1^ and LFOD 296 (293 to 298) mOsmol·kg^−1^ and 297 (293 to 301) mOsmol·kg^−1^, respectively], or exercise-induced BM loss [HFOD 1.7 (1.2 to 2.2) % and HFOD 2.2 (1.6 to 2.7) %] was observed between the two FODMAP dietary trials in response to EHS.

### 3.2. Plasma Microbial Concentration

**Figure 2:**
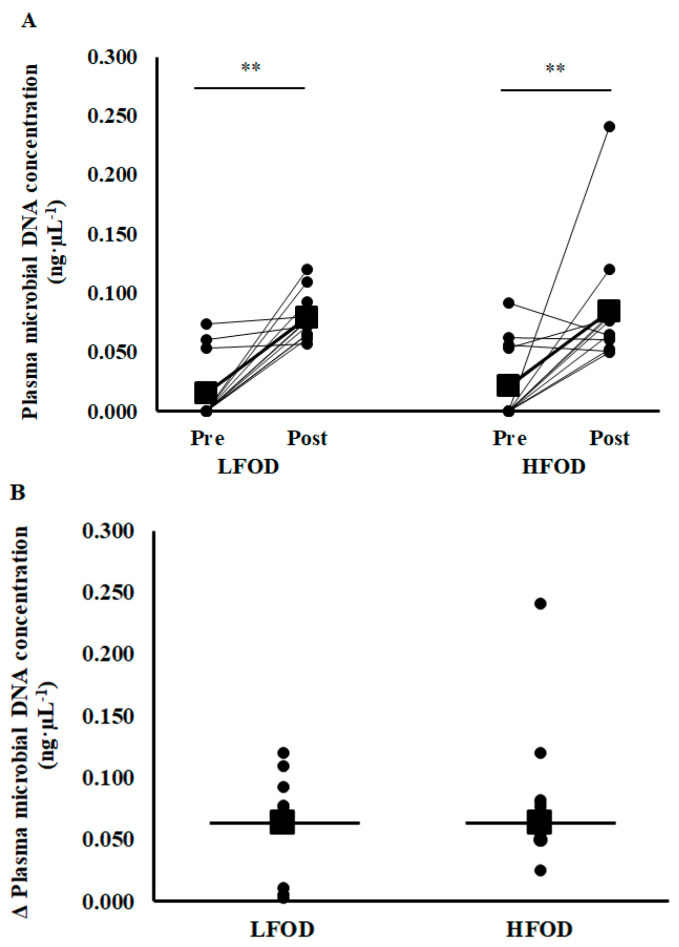
Total plasma microbial DNA absolute content (**A**) and relative change (**B**) in response to exertional-heat stress (EHS). Mean (■) and individual responses (•) (*n* = 13): ** *p* < 0.01 pre- to post-EHS difference.

Resting pre-EHS total plasma microbial DNA concentration did not differ between the two FODMAP dietary trials (Figure 2A). Total plasma microbial DNA significantly increased (*p* < 0.001) pre- to post-EHS in LFOD and HFOD. No significant difference in EHS-associated Δ in plasma microbial DNA concentration between LFOD and HFOD was observed (Figure 2B).

### 3.3. Plasma Bacteria α-Diversity and Relative Abundance

Relative abundance of *Archaea* and *Eukaryota* were detected at 0.035% and 0.001%, respectively. The bacterial α-diversity (SEI) and predominance for phyla, family, and genus bacterial groups in plasma are depicted in Table 1. No difference in SEI and absolute relative abundance of bacterial phyla groups was observed pre- and post-EHS between LFOD and HFOD. There was no significant difference observed for pre- to post-EHS plasma bacterial phyla relative abundance on LFOD and HFOD or for EHS Δ in bacterial phyla relative abundance between LFOD and HFOD (Table 1). No difference in SEI and absolute relative abundance of bacterial family groups was observed pre- and post-EHS between LFOD and HFOD. There was no significant difference observed for pre- to post-EHS plasma bacterial family relative abundance on LFOD and HFOD or for EHS Δ in bacterial family relative abundance between LFOD and HFOD (Table 1). No difference in SEI and absolute relative abundance of bacterial genus groups was observed pre- and post-EHS between LFOD and HFOD. There was a significant difference observed for pre- to post-EHS for *Delftia* and *Serratia* bacterial genus relative abundance on LFOD, for *Bacillus* bacterial genus relative abundance on HFOD, and for EHS Δ in bacterial family relative abundance between LFOD and HFOD (Table 1).

### 3.4. Fecal Microbial Taxa

The bacterial taxa α-diversity (SEI) and predominance for phyla, family, and genus bacterial groups in feces are depicted in Table 2. At rest, the sufficient identification of relative abundance of bacterial phyla groups in fecal samples included *Firmicutes*, *Bacteroidota*, *Proteobacteria*, *Actinobacteroita*, and *Verrucomicrobia*. Resting fecal bacterial phyla SEI on LFOD and HFOD did not significantly differ (*p* = 0.136, *d* = 0.48). LFOD had significantly higher *Firmicutes* (*p* = 0.017) and lower *Bacteroidota* (*p* = 0.011) compared with HFOD. The sufficient identification of relative abundance of bacterial family groups in fecal samples on both dietary interventions included *Ruminococcaceae*, *Lachnospiraceae*, *Bacteroidaceae*, *Prevotellaceae*, *Acidaminococcaceae*, *Enterobacteriaceae, Akkermansiaceae*, *Peptostreptococcaceae*, *Rikenellaceae*, and *Christensenellaceae*, with all other bacterial family groups <2% relative abundance. Resting fecal bacterial family SEI on LFOD and HFOD did not substantially differ but showed a trend for higher diversity on HFOD (*p* = 0.093, *d* = 0.76). The relative abundance of *Ruminococcaceae* (*p* = 0.025) was significantly higher on LFOD compared to HFOD. The sufficient identification of relative abundance of bacterial genus groups in fecal samples on both dietary interventions included *Faecalibacterium*, *Bacteroides*, *Roseburia*, *Subdoligranulum*, *Phascolarctobacterium*, *Blautia*, *Ruminococcaceae UCG-002*, *Prevotella 9*, *Akkermansia*, *Escherichia-Shigella*, *Agathobacter*, *Christensenellaceae R-7 group*, and *Alistipes*, with all other bacterial family groups >2% relative abundance. Resting fecal bacterial genus SEI on LFOD and HFOD did not substantially differ (*p* = 0.676, *d* = 0.10). No significant difference in the relative abundance of bacterial genus groups was observed between LFOD and HFOD, likely due to large individual variations in population numbers. However, individual observations showed *Prevotellaceae NK3B31* was trace on LFOD (>0.1%), but 2.6% on HFOD, respectively. Conversely, *Escherichia-Shigella* was trace on HFOD (>0.1%), but 6.3% on LFOD.

### 3.5. Plasma and Fecal Short Chain Fatty Acid Concentrations

**Figure 3:**
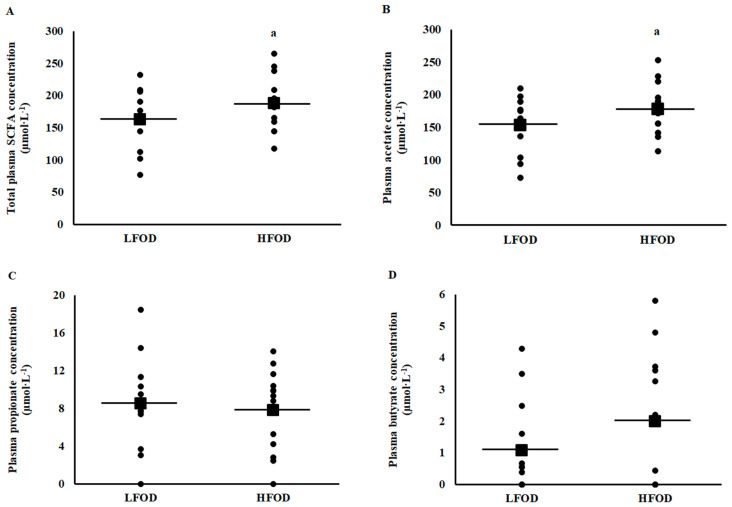
Pre-EHS plasma total short chain fatty acid (**A**), acetate (**B**), propionate (**C**), and butyrate (**D**) concentrations. Mean (■) and individual responses (•) (*n* = 13): ^a^ *p* < 0.05 LFOD and HFOD difference.

**Figure 4:**
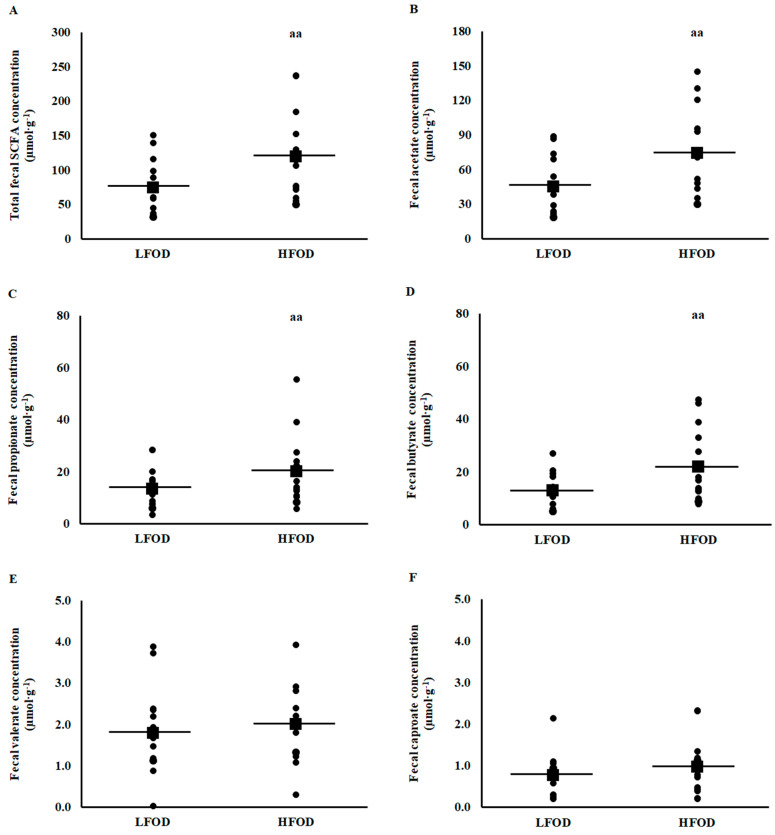
Pre-EHS fecal total short chain fatty acid (**A**), acetate (**B**), propionate (**C**), butyrate (**D**), valerate (**E**), and caproate (**F**) concentrations. Mean (■) and individual responses (•) (*n* = 13): ^aa^ *p* < 0.01 LFOD and HFOD difference.

Resting pre-EHS total plasma SCFA (*p* = 0.04; *d* = 0.47) and plasma acetate (*p* = 0.036, *d* = 0.50) concentrations, but not plasma propionate (*p* = 0.239) and butyrate (*p* = 0.646) concentrations, were significantly higher on HFOD compared with LFOD (Figure 3). Resting pre-EHS fecal total SCFA (*p* < 0.001; *d* = 0.75), acetate (*p* = 0.001, *d* = 0.78), propionate (*p*= 0.011; *d* = 0.53), and butyrate (*p* = 0.002; *d* = 0.77) concentrations, but not valerate (*p* = 0.074) and caproate (*p* = 0.203) concentrations, were significantly higher on HFOD compared with LFOD (Figure 4). No difference in resting pre-EHS total and differential fecal branched-chain fatty acid (i.e., iso-butyric and iso-valeric acids) concentrations were observed between LFOD and HFOD. However, a significantly (*p* = 0.005; *d* = 0.70) lower total BSCFA:SCFA ratio was observed on HFOD [4.2 (2.5 to 5.3) %] compared with LFOD [7.7 (4.4 to 9.5) %].

## 4. Discussion

The aims of the current study were to explore the impact of EHS on the magnitude and profile of circulatory whole bacteria profile, and to determine the impact of FODMAP dietary intervention on this magnitude and profile in response to EHS. In accordance with our hypothesis, 2 h of EHS induced bacteremia as indicated by pre- to post-EHS increases in plasma microbial DNA concentration, characterized by increased *Proteobacteria* family and genus groups. On the contrary to our hypothesis, no substantial difference in absolute and relative change in pre- and post-EHS plasma microbial DNA concentration was observed between FODMAP dietary intervention trials. These outcomes were observed despite the 24 h FODMAP dietary interventions (i.e., eucaloric low (2 g·day^−1^) and high (51 g·day^−1^) FODMAP content) resulting in a substantial difference in pre-EHS fecal and plasma bacterial relative abundance, which likely promoted the substantial difference in fecal (i.e., acetate, butyrate, and propionate) and plasma (i.e., acetate) SCFA concentrations between FODMAP trials. The authors acknowledge the lack of pre-dietary intervention sample collection and variable analysis, established as part of the original experimental design [11], and consider this a limitation in the current study. However, the previous identification of test–retest mirrored verification and/or confirmed reliability provides a strong base to suggest that the FODMAP content of a 24 h dietary intake may have the potential to alter fecal [20] and, in addition to the findings of the current study, plasma SCFA concentrations [20]. From a translational perspective, the current results highlight the presence of a systemic microbiome, as previously suggested in the clinical arena [33], and highlight the changes that occur in response to substantial exposure to EHS. Admittedly, the positive or negative outcomes of such haematological change, indicative of a sub-level sepsis, on health or performance outcomes are not clear and warrant further exploration through appropriate methodological applications (e.g., sufficient exertional stress, with or without heat exposure). Tt is, however, a likely contributor to the commonly reported exercise-associated systemic inflammatory responses of EIGS and subsequent clinical consequences [29,30,31]. As such, although FODMAP dietary intervention was not successful as a prevention and management strategy for EHS associated bacteremia, future research should explore other means of ameliorating the observed EIGS pathophysiology.

### 4.1. Plasma Microbial DNA Concentration

A novel aspect of the current study was the exploration of the ‘blood microbiome’ at rest and in response to EHS. The results suggest that exposure to EHS promoted an increase in whole bacterial DNA concentration in circulation (i.e., Δ 0.064 ng·μL^−1^), as measured by total microbial DNA extraction and subsequent fluorometric quantification. These results are supported by a previous test–retest experimental procedure using a HIIT exercise protocol (i.e., 6 × 20 min exercise block consisting of 210 s running at 55–60% $\dot{V}$O_2max_, 60 s running at 65–70% $\dot{V}$O_2max_, and 30 s running at 80–85% $\dot{V}$O_2max_, followed by 20 plyometric jumps of alternative legs repeated three times per exercise block), reporting a Δ pre- to post-exercise microbial DNA concentration of 0.040 ng·μL^−1^ [20]. The current EHS model presented high Δ microbial DNA concentration compared to the previous HIIT exercise protocol, which is not surprising considering the additional heat exposure that contributes to a potent exacerbation factor in perturbing intestinal epithelial integrity, as per EIGS pathophysiology [12]. The authors are aware of and acknowledge the potential confounding contribution of exercise-induced cellular damage-free DNA to the observed values [61] and consider this a potential study limitation. It is, however, important to highlight that this misinterpretation risk is overcome by the inclusion of bacteria composition profile determination, and the acknowledgement of interpreting results of microbial concentration and bacterial profile together. The plasma microbial DNA outcomes are supported by the observed increase in the relative abundance of *Proteobacteria* and *Firmicutes* phylum bacterial groups in response to the current EHS, as well as the increased relative abundance of *Proteobacteria* in a previous exertional stress experimental model [20]. This suggests that some instigation of exercise-associated bacteremia is occurring. The mechanisms to explain these outcomes are likely attributed to the unrestricted paracellular translocation of intestinal lumen originating whole bacteria into mainstream circulation as a result of intestinal epithelial cellular damage (i.e., membrane rupture and/or full cellular destruction) [62,63]. Indeed, such cellular damage and pathogenic translocation is commonly reported with enterocyte injury and/or bacterial endotoxin determination (i.e., I-FABP, direct LPS, and/or surrogate markers sCD14, LBP, and/or anti-endotoxin antibodies) due to the EIGS circulatory–gastrointestinal pathway, linked to exercise-associated splanchnic hypoperfusion and subsequently gastrointestinal ischemia [64,65,66,67]. These pathophysiology pathways appear to be exacerbated with the inclusion of substantial (i.e., ≥35 °C) heat exposure [12]. Nevertheless, such pathogenic luminal translocation into internal body circulation has the potential to activate systemic immune responses, namely polymorphonuclear leukocytes and/or inflammatory cytokine responses, in its attempt to deal with the foreign onslaught [8,13,14,15,27,28], which may lead to clinical implications (e.g., systemic inflammatory response syndrome) [29,30,31]. Therefore, prevention and management strategies to mitigate such EHS bacteremia appears warranted and essential. From this perspective, previous research has reported a differential effect of dietary FODMAP content on the surrogate markers of the EIGS circulatory–gastrointestinal pathway, with a 24 h high FODMAP dietary intervention showing lower EHS induced intestinal epithelial injury (i.e., plasma I-FABP concentration) and modest bacterial endotoxin translocation outcomes (i.e., plasma LBP and sCD14), but no difference in systemic inflammatory cytokine responses (i.e., plasma IL-1β, TNFα, IL-6, IL-8, and IL-10 concentrations), compared with a 24 h high FODMAP dietary intervention [11]. Results from the current study add to these previous findings and suggest the dietary FODMAP of the preceding 24 h diet does not influence EHS-associated bacteremia. Considering the EHS is in accordance with experimental models able to promote EIGS pathophysiology of clinical significance [3], it is likely that the mechanisms by which a 24 h high FODMAP may provide some protection (i.e., splanchnic perfusion and SCFA) were not potent enough on this occasion to warrant any substantial beneficial effect, as per the modest attenuation seen on EHS endotoxemia in the original study [11]. It is clear that future research needs to establish whether more potent EIGS prevention and management strategies, such as pre- and during-exercise feeding [17], provide greater benefits in the prevention or management of EHS bacteremia.

### 4.2. Plasma Bacterial Profile

An interesting finding in the current study was the predominant type of potential bacterial translocation that occurred. The predominant phylum in resting pre-EHS plasma samples was similar to those previously reported in clinical research, whereby *Proteobacteria*, *Firmicutes*, and *Actinobacteria* are consistently observed to be predominant plasma bacterial phyla [33]. The current study took an additional step in exploring the bacterial family and genus using standardised 16S rRNA amplicon sequencing methodologies (Table 1) and observed predominant family (i.e., *Pseudomonadaceae*, *Yersiniaceae*, *Comamonadaceae*, and *Sphingomonadaceae*) and genus (i.e., *Pseudomas*, *Serrata*, *Delftia*, and *Sphingomonas*) from the *Proteobacteria* bacterial phylum group. It was clear that the α-diversity and relative abundance of phyla, family, and genus was far lower than the outcomes from the faecal sample determination of bacterial composition, which is not surprising considering the more stringent barrier and immune regulation for systemic circulation entry versus the gastrointestinal lumen entry. With the exception of the bacterial phyla relative abundance profile of *Proteobacteria-Pseudomonadaceae* and *Proteobacteria-Pseudomas*, the resting pre-EHS α-diversity and relative abundance of these bacterial groups substantially differed from those previously reported in a recreational athlete population prior to a 2 h HIIT protocol on two separate occasions [20]. Considering the test–retest repeatability for SEI reported in Young et al. [20], this inconsistency suggests a large variation between individuals at the lower level of bacterial profiling, warranting caution when interpreting a standardised resting ‘blood microbiome’ profile, within a healthy active population that is not immunocompromised and does not present acute systemic infection. Such findings raise further questions, such as, ‘what is the possibility of bacteria coexisting in systemic circulation as part of health homeostasis, and does regular physical activity alter this profile’? ‘Whether the blood microbiome has implications within EIGS pathophysiology’? Or ‘are these observations simply inconsequential and tightly regulated by immune surveillance’?

In response to EHS, and noting the increase in plasma microbial concentration, changes to the ‘blood microbiome’ profile were observed. Although the α-diversity (SEI) did not change between pre- and post-EHS and was not different between trials. Subtle changes in the blood bacterial profile were apparent, namely an increase in the relative abundance of *Proteobacteria* (+1.6%) and *Firmicutes* (+0.6%). An interesting observation was the trend for a greater EHS-associated increase in *Proteobacteria* relative abundance on HFOD (+3.0%) compared with LFOD (+0.3%) (*p* = 0.055). This result may suggest a greater alteration impact of HFOD on plasma bacteria profile. However, this is not clear and warrants further exploration, especially due to the pathogenicity of *Proteobacteria* bacterial groups [68], if these changes and/or trial differences are due to luminal translocation or alterations in systemic immune surveillance and/or functional responses. Moreover, future exploration needs to consider individual variation and characteristics that may impact plasma bacterial profile, such as recurrent Ex-GIS possibly aligned with fitness status and biological sex [7,69,70], which have been identified as potential exacerbation factors in EIGS pathophysiology [4]. These outcomes are different from a previous test–retest 2 h HIIT experimental model reporting increases in the relative abundance of *Proteobacteria* and respective family and genus bacterial groups but decreases in *Firmicutes* [20]. Such inconsistencies in exercise-associated changes to the relative abundance of plasma family and genus bacterial groups highlights the large inter individual variation in biomarkers and/or variables associated with extrinsic stimuli (e.g., exercise intensity and magnitude of heat exposure). Although it is of scientific interest to observe and explore the blood bacterial profile and change induced by external stimuli (e.g., EHS), it would appear the overall bacterial load (i.e., plasma microbial DNA concentration) may override the bacterial profile (i.e., type of bacteria present) in its potency in implicating clinical issues in the population liable to bacteremia. However, the authors acknowledge the scientific interest in plasma bacterial profile observation, but understand the current sample size was calculated *a priori* for the original EIGS and FODMAP intervention research [11], which may be considered a limitation to the current study. Thus, the current study appears to be underpowered for any concrete plasma bacterial profile change definitive conclusions. The post hoc power calculation supports the plasma microbial DNA concentration change, but not bacterial profile; therefore, caution is warranted when extrapolating such plasma bacterial profile status and changes into the practical arena in the prevention or management of EIGS.

### 4.3. Resting Pre-EHS Fecal Bacterial Profile

To provide further insight and explanation into the findings of total plasma microbial DNA concentration and bacterial profile in response to EHS, faecal bacterial taxa was also determined (Table 2). In accordance with previous exercise stress research models [7,20], the resting fecal bacterial α-diversity and relative abundance was similar and characterised by predominant *Firmicutes* (69.3%) and *Bacteroidota* (17.5%), followed by *Proteobacteria* (5.3%) and *Actinobacteroita* (4.1%) relative abundance, including similar predominant family (i.e., *Ruminococcaceae*, *Lachnospiraceae*, *Bacteroidaceae*, and *Prevotellaceae*) and genus (i.e., *Bacteroides*, *Faecalibacterium*, *Subdoligranulum*, *Blautia*, *Phascolarctobacterium*, *Ruminococcaceae UCG-002*, and *Prevotella-9*) bacterial groups [7,20]. The FODMAP content of a provided 24 h diet was sufficient to result in differences in resting fecal bacterial profile. For example, LFOD resulted in substantially and significantly higher resting fecal *Firmicutes* (73.9%), *Bacteroidota* (9.7%), and *Ruminococcaceae* (34.2%) relative abundance compared with HFOD (64.6%, 25.3%, and 26.1%; respectively). Also noteworthy, *Escherichia-Shigella* was trace on HFOD (>0.1%), but substantially higher on LFOD (6.3%). With the fecal bacterial profile in mind, the similarities in total plasma microbial DNA concentration and differences in plasma bacterial profile between LFOD and HFOD are unlikely to be due to the larger differences in faecal bacterial taxa observed between the two dietary interventions. Therefore, short-term gastrointestinal microbiota changes induced by dietary challenge does not appear to influence to any large extent the systemic bacterial concentration or profile.

### 4.4. Fecal and Plasma Short Chain Fatty Acids

A strategy that is gaining interest, in regard to the prevention and management of EIGS, is the manipulation of the gastrointestinal microbial composition via intervention (i.e., dietary or nutritional supplementation) in order to increase the relative abundance of commensal bacteria with ability for SCFA production and decrease the relative abundance of potential pathogenic microbes and associated endotoxins [7,34,43]. It is suggested that dietary manipulation, such as increasing fermentable carbohydrates that principally act as prebiotics, may subsequently lead to increased SCFA commensal bacterial diversity and SCFA luminal concentration [35,36,37]. Such alterations in luminal composition may provide a protective mechanism to intestinal epithelial integrity and systemic responses, although the focus has been primarily in clinical populations [38,39,40]. For example, in the current study, the fecal bacterial profile differed between LFOD and HFOD, which likely contributed to the substantial differences in fecal total and differential SCFA concentrations observed between the FODMAP interventions. An additional interesting finding was that these luminal changes appear to also have impacted plasma total and acetate concentrations but not plasma butyrate and propionate concentrations. This suggests that the FODMAP content of a 24 h diet can influence, to some extent, the translocation of acetate, but not butyrate or propionate, into circulation, noting that the absolute acetate concentration was far greater than the other SCFA, which may have contributed to the observed plasma SCFA outcomes [71]. As previously mentioned, the authors acknowledge the study limitation of not collecting and analysing a pre-dietary intervention sample, due to the original experimental design focusing on circulatory–gastrointestinal pathway markers of EIGS [11]. However, the previous identification of test–retest mirrored verification and/or confirmed reliability provides a strong base to suggest that the FODMAP content of a 24 h dietary intake may have the potential to alter fecal and plasma SCFA concentrations [20].

Both the lumen and plasma level of SCFA appear to be important for the management of the circulatory–gastrointestinal pathway of EIGS considering the negative association observed between these levels and EIGS biomarkers of gastrointestinal integrity status [11,20,72]. In accordance with mechanisms of action previously proposed as to why FODMAP intake prior to exercise may be protective in attenuating intestinal epithelial injury and endotoxemia [11], the increase in lumen and circulating SCFA associated with a 24 h high FODMAP may contribute to attenuating gastrointestinal integrity (the circulatory–gastrointestinal pathway of EIGS) perturbations. From a practical translation perspective, a high FODMAP diet is synonymous with promoting Ex-GIS severity due to bacterial fermentation of poorly absorbed sugars that have been consumed and the increased lumen content (CO_2_, H_2_, CH_4_, N_2_, and/or water) as part of the fermentation process. However, the increase in lumen and plasma SCFA concentration may provide some mechanistic explanation for the EIGS-attenuating effects of high FODMAP dietary intake [11,72]. From a bacteremia perspective, although there exists evidence for high FODMAP content in the diet providing some potential up-stream EIGS protective effect [11], it appears that FODMAP content provided 24 h before EHS does not appear to have any influential effect and therefore cannot be considered a clear prevention and management strategy for EHS-associated bacteremia.

## 5. Conclusions

Substantial exposure to EHS can induce bacteraemia as indicated by a pre- to post-EHS increase in total microbial DNA concentration in systemic circulation, characterised by increased *Proteobacteria* phyla, family, and genus groups. No difference in absolute and relative change in pre- and post-EHS microbial DNA concentration was observed between FODMAP dietary intervention trials. These outcomes were observed despite the 24 h FODMAP dietary interventions resulting in substantial different pre-EHS faecal bacterial relative abundance, which likely promoted the substantial difference in faecal (i.e., acetate, butyrate, and propionate) and plasma (i.e., acetate only) SCFA concentrations between FODMAP trials.

## Figures and Tables

**Table 1:** Relative abundance (% RA) of plasma bacterial phylum, family, and genus AVS at rest prior to exertional-heat stress (pre-EHS) and in response to EHS (post-EHS).

		LFOD			HFOD	
	Pre-EHS	Post-EHS	Δ	Pre-EHS	Post-EHS	Δ
**Phylum**						
SEI	0.126 (0.036)	0.121 (0.029)	−0.005 (0.048)	0.129 (0.033)	0.122 (0.038)	−0.007 (0.047)
*Actinobacteriota*	2.309 (1.187)	3.042 (2.571)	0.734 (2.726)	1.874 (1.285)	2.357 (1.493)	0.483 (1.786)
*Bacteroidota*	1.181 (1.537)	0.641 (0.904)	−0.540 (1.899)	1.192 (1.480)	0.630 (1.097)	−0.562 (2.143)
*Cyanobacteria*	1.310 (1.853)	0.422 (0.556)	−0.888 (1.965)	1.393 (3.394)	0.912 (1.358)	−0.481 (3.915)
*Firmicutes*	6.142 (2.004)	6.801 (2.384)	0.659 (2.511)	6.669 (3.443)	7.249 (3.175)	0.580 (5.069)
*Proteobacteria*	87.480 (4.951)	87.772 (4.057)	0.293 (4.898)	84.796 (6.052)	87.763 (4.402)	2.967 (6.520)
**Family**						
SEI	0.228 (0.030)	0.244 (0.017)	−0.004 (0.037)	0.237 (0.035)	0.231 (0.028)	−0.012 (0.035)
*Actinobacteriota-*						
*Propionibacteriaceae*	0.662 (0.612)	0.343 (0.330)	−0.320 (0.614)	0.689 (0.519)	0.609 (0.431)	−0.80 (0.557)
*Cyanobacteria-*						
*Chloroplast*	1.169 (1.883)	0.333 (0.539)	−0.836 (2.071)	1.349 (3.409)	0.912 (1.358)	−0.437 (3.917)
*Firmicutes-*						
*Bacillaceae*	1.928 (1.131)	2.585 (1.651)	0.657 (1.807)	1.525 (1.397)	2.246 (1.095)	0.721 (1.552)
*Carnobacteriaceae*	2.083 (1.322)	2.368 (1.429)	0.285 (1.286)	3.261 (2.477)	2.947 (1.395)	−0.314 (3.351)
*Streptococcaceae*	0.518 (1.024)	0.367 (0.436)	−0.151 (1.121)	0.522 (0.718)	0.761 (0.956)	0.239 (1.212)
*Staphylococcaceae*	0.759 (0.960)	0.635 (0.492)	−0.125 (1.251)	0.424 (0.549)	0.494 (1.285)	0.070 (1.449)
*Proteobacteria-*						
*Beijerinckiaceae*	0.380 (0.591)	0.481 (0.524)	0.101 (0.810)	0.725 (1.087)	0.672 (0.882)	−0.054 (1.374)
*Xanthobacteraceae*	2.550 (1.448)	1.857 (1.439)	−0.693 (2.421)	2.881 (2.466)	2.240 (1.602)	−0.641 (2.460)
*Sphingomonadaceae*	4.694 (2.832)	4.668 (2.700)	−0.026 (2.076)	4.717 (3.919)	3.597 (2.830)	−1.120 (3.538)
*Comamonadaceae*	5.093 (2.120)	6.649 (1.722)	1.556 (2.687)	6.317 (2.910)	6.533 (2.893)	0.215 (4.256)
*Oxalobacteraceae*	1.327 (1.371)	0.815 (0.663)	−0.513 (1.711)	1.432 (1.746)	0.766 (0.651)	−0.666 (1.711)
*Yersiniceae*	15.472 (3.586)	15.269 (3.653)	−0.203 (6.069)	14.003 (4.061)	16.371 (2.799)	2.368 (4.183)
*Moraxellaceae*	0.493 (0.577)	0.329 (0.478)	−0.164 (0.636)	0.936 (1.157)	0.250 (0.298)	−0.685 (1.208)
*Pseudomonadaceae*	54.430 (7.778)	54.878 (4.975)	0.447 (7.280)	50.249 (6.352)	53.475 (6.400)	3.226 (9.089)
**Genus**						
SEI	0.195 (0.018)	0.198 (0.015)	0.003 (0.023)	0.201 (0.032)	0.201 (0.025)	−0.001 (0.045)
*Actinobacteriota-*						
*Cutibacterium*	0.628 (0.635)	0.272 (0.256)	−0.356 (0.726)	0.678 (0.510)	0.572 (0.399)	−0.106 (0.508)
*Firmicutes-*						
*Bacillus*	1.551 (1.010)	2.364 (1.500)	0.813 (1.603)	1.173 (1.284)	1.884 (0.904) *	0.711 (1.358)
*Carnobacterium*	2.083 (1.322)	2.366 (1.428)	0.284 (1.283)	3.170 (2.434)	2.705 (1.349)	−0.465 (3.286)
*Streptococcus*	0.518 (1.024)	0.272 (0.393)	−0.246 (1.061)	0.522 (0.718)	0.761 (0.956)	0.239 (1.212)
*Staphylococcus*	0.644 (0.735)	0.635 (0.492)	−0.009 (1.065)	0.424 (0.549)	0.491 (1.286)	0.066 (1.449)
*Proteobacteria-*						
*Methylobacterium*	0.355 (0.541)	0.362 (0.459)	0.007 (0.654)	0.725 (1.087)	0.672 (0.882)	−0.054 (1.374)
*Sphingomonas*	4.268 (2.608)	4.522 (2.617)	0.255 (2.074)	4.430 (4.030)	3.388 (2.898)	−1.042 (3.440)
*Delftia*	4.626 (2.119)	6.625 (1.758) *	1.999 (2.854)	5.553 (2.618)	6.342 (2.756)	0.789 (4.062)
*Massilia*	1.280 (1.362)	0.809 (0.666)	−0.472 (1.686)	1.432 (1.746)	0.709 (0.667)	−0.723 (1.710)
*Serratia*	6.374 (1.437)	4.903 (1.508) *	−1.472 (2.783)	5.303 (2.140)	6.143 (2.068)	0.840 (3.005)
*Pseudomonas*	54.430 (7.778)	54.878 (4.975)	0.447 (7.280)	50.249 (6.352)	53.475 (6.400)	3.226 (9.089)

Mean (SD) of ≥0.5% RA (*n* = 13): * *p* < 0.05 vs. pre-EHS. AVS: amplicon sequence variant, EHS: exertional-heat stress, HFOD: high fermentable oligo- di- mono-saccharides and polyol diet, LFOD: low fermentable oligo- di- mono-saccharides and polyol diet, RA: relative abundance, SEI: Shannon Equitability Index.

**Table 2:** Relative abundance (%) of fecal bacterial phylum, family, and genus AVS at rest prior to exertional-heat stress (pre-EHS).

	LFOD	HFOD
**Phylum**		
SEI	0.197 (0.070)	0.225 (0.056)
*Actinobacteriota*	2.573 (1.451)	5.542 (5.303)
*Bacteroidota*	9.707 (4.486) ^a^	25.347 (16.395) ^a^
*Firmicutes*	73.854 (20.057) ^a^	64.648 (14.769) ^a^
*Proteobacteria*	9.075 (18.101)	1.472 (2.640)
*Verrucomicrobia*	3.905 (9.722)	2.674 (3.183)
**Family**		
SEI	0.256 (0.034)	0.284 (0.028)
*Actinobacteriota*		
*Bifidobacteriaceae*	0.483 (0.509)	1.886 (2.423)
*Coriobacteriaceae*	1.261 (1.154)	2.637 (3.149)
*Eggerthellaceae*	0.511 (0.380)	0.879 (0.884)
*Bacteroidota*		
*Bacteroidaceae*	5.305 (4.418)	8.742 (7.952)
*Barnesiellaceae*	0.546 (0.851)	1.264 (1.653)
*Muribaculaceae*	0.211 (0.356)	1.416 (1.856)
*Prevotellaceae*	1.917 (2.322)	9.230 (9.755)
*Rikenellaceae*	1.217 (0.923)	3.768 (4.131)
*Tannerellaceae*	0.360 (0.300)	0.616 (0.487)
*Firmicutes*		
*Streptococcaceae*	0.155 (0.188)	3.420 (7.152)
*Christensenellaceae*	3.353 (2.773)	1.374 (1.216)
*Clostridiaceae 1*	1.162 (1.033)	0.325 (0.403)
*Lachnospiraceae*	24.878 (12.126)	24.697 (10.165)
*Peptostreptococcaceae*	3.816 (6.336)	1.154 (1.323)
*Ruminococcaceae*	34.221 (9.095) ^a^	26.057 (8.994) ^a^
*Acidaminococcaceae*	4.226 (5.596)	5.384 (8.562)
*Veillonellaceae*	1.152 (1.264)	1.531 (2.323)
*Proteobacteria*		
*Enterobacteriaceae*	8.377 (18.253)	0.033 (0.062)
*Pasteurellaceae*	0.486 (0.844)	1.139 (2.724)
*Verrucomicrobia*		
*Akkermansiaceae*	3.905 (9.722)	2.673 (3.183)
**Genus**		
SEI	0.298 (0.041)	0.298 (0.028)
*Actinobacteriota*		
*Bifidobacterium*	0.480 (0.170)	1.886 (0.857)
*Collinsella*	1.257 (0.384)	2.637 (1.113)
*Bacteroidota*		
*Bacteroides*	5.305 (1.473)	8.742 (2.811)
*Barnesiella*	0.533 (0.279)	1.184 (0.584)
*Prevotella-9*	1.081 (0.506)	6.288 (3.058)
*Prevotellaceae-NK3B31*	0.032 (0.021)	2.553 (2.451)
*Alistipes*	1.090 (0.245)	3.758 (1.458)
*Firmicutes*		
*Streptococcus*	0.153 (0.063)	3.407 (2.531)
*Christensenellaceae R-7*	3.335 (0.924)	1.351 (0.430)
*Clostridium sensu stricto-1*	1.161 (0.344)	0.322 (0.141)
*Agathobacter*	3.274 (1.191)	2.150 (1.141)
*Anaerostipes*	0.617 (0.241)	1.493 (0.689)
*Blautia*	3.460 (0.820)	5.400 (2.022)
*Coprococcus-2*	0.771 (0.277)	0.395 (0.157)
*Coprococcus-3*	0.627 (0.160)	0.657 (0.252)
*Dorea*	1.408 (0.284)	1.125 (0.367)
*Fusicatenibacter*	1.008 (0.357)	1.063 (0.650)
*Lachnospiraceae-NK4A136*	1.722 (0.580)	0.604 (0.268)
*Roseburia*	6.056 (1.971)	3.692 (1.072)
*Eubacterium-eligens*	0.414 (0.164)	1.732 (1.306)
*Eubacterium-hallii*	0.662 (0.163)	1.036 (0.353)
*Ruminococcus-torques*	1.016 (0.395)	0.919 (0.284)
*Intestinibacter*	2.125 (1.857)	0.322 (0.248)
*Romboutsia*	1.245 (0.447)	0.759 (0.309)
*Faecalibacterium*	13.505 (3.900)	6.647 (3.359)
*Ruminiclostridium-6*	0.485 (0.217)	0.884 (0.598)
*Ruminococcaceae-NK4A214*	0.990 (0.454)	1.385 (0.473)
*Ruminococcaceae-UCG-002*	3.612 (0.768)	4.329 (1.717)
*Ruminococcaceae-UCG-010*	0.714 (0.302)	0.291 (0.137)
*Ruminococcaceae-UCG-014*	1.715 (0.496)	1.492 (0.883)
*Ruminococcus-1*	0.994 (0.493)	1.201 (0.589)
*Ruminococcus-2*	1.396 (0.649)	0.630 (0.355)
*Subdoligranulum*	5.584 (2.869)	4.045 (1.278)
*Eubacterium-coprostanoligenes*	1.757 (0.479)	1.508 (0.523)
*Phascolarctobacterium*	4.129 (1.874)	5.384 (3.027)
*Veillonella*	0.746 (0.297)	0.260 (0.115)
*Proteobacteria*		
*Escherichia-Shigella*	6.263 (5.913)	0.006 (0.004)
*Klebsiella*	1.671 (1.339)	0.025 (0.021)
*Haemophilus*	0.461 (0.281)	0.975 (0.801)
*Verrucomicrobia*		
*Akkermansia*	3.905 (3.241)	2.673 (1.125)

Mean (SD) of ≥0.5% RA (*n* = 9): ^a^ *p* < 0.05 LFOD and HFOD difference. AVS: amplicon sequence variant, EHS: exertional-heat stress, HFOD: high fermentable oligo- di- mono-saccharides and polyol diet, LFOD: low fermentable oligo- di- mono-saccharides and polyol diet, RA: relative abundance, SEI: Shannon Equitability Index.

## Data Availability

The data presented in this study are available upon reasonable request from the corresponding author. The data are not publicly available due to privacy and confidentiality of data in accordance with ethics committee approve procedures.
